# Feasibility study of structural similarity index for patient‐specific quality assurance

**DOI:** 10.1002/acm2.14591

**Published:** 2024-12-03

**Authors:** Jae Choon Lee, Hyeong Wook Park, Young Nam Kang

**Affiliations:** ^1^ Department of Medical Physics Kyonggi University Suwon South Korea; ^2^ Department of Radiation Oncology Seoul St. Mary's Hospital, College of Medicine The Catholic University of Korea Seocho‐gu Seoul South Korea

**Keywords:** gamma evaluation, patient‐specific quality assurance, Pearson correlation coefficient, structural similarity algorithm

## Abstract

**Background:**

The traditional gamma evaluation method combines dose difference (DD) and distance‐to‐agreement (DTA) to assess the agreement between two dose distributions. However, while gamma evaluation can identify the location of errors, it does not provide information about the type of errors.

**Purpose:**

The purpose of this study is to optimize and apply the structural similarity (SSIM) index algorithm as a supplementary metric for the quality evaluation of radiation therapy plans alongside gamma evaluation. By addressing the limitations of gamma evaluation, this study aims to establish clinically meaningful SSIM criteria to enhance the accuracy of patient‐specific quality assurance (PSQA).

**Methods:**

We analyzed the relationship between the gamma passing rate (GPR) and the SSIM index with respect to distance and dose errors. For SSIM analysis corresponding to gamma evaluation criteria of 3%/2 mm, we introduce the concept of SSIM passing rate (SPR). We determined a valid SSIM index that met the gamma evaluation criteria and applied it. Evaluations performed for 40 fields measured with an electronic portal imaging device (EPID) were analyzed using the GPR and the applied SPR.

**Results:**

The study results showed that distance errors significantly affected both the GPR and the SSIM index, whereas dose errors had some influence on the GPR but little impact on the SSIM index. The SPR was 100% for distance error of 2 mm but began to decrease for distance errors of 3 mm or more. An optimal SSIM index threshold of 0.65 was established, indicating that SPR fell below 100% when distance errors exceeded 2 mm.

**Conclusions:**

This study demonstrates that the SSIM algorithm can be effectively applied for the quality evaluation of radiation therapy plans. The SPR can serve as a supplementary metric to gamma evaluation, offering a more precise identification of distance errors. Future research should further validate the efficacy of SSIM algorithm across a broader range of clinical cases.

## 1 INTRODUCTION

In radiation therapy, the delivery of precise doses to patients is a crucial challenge. Patient‐specific quality assurance (PSQA) is essential for ensuring that the dose delivered to the patient aligns with the treatment plan generated by the treatment planning system (TPS).^1^, ^2^, ^3^, ^4^, ^5^, ^6^, ^7^, ^8^ Various analytical methods, such as dose difference (DD), distance‐to‐agreement (DTA), and gamma evaluation, have been developed to quantitatively assess the consistency between planned and delivered doses.^9^ DD is suitable for areas with a shallow dose gradient but becomes highly sensitive in regions with a steep dose gradient. Conversely, DTA performs well in areas with steep dose gradients but may produce high values in regions with a shallow dose gradient. Gamma evaluation combines the concepts of DD and DTA to calculate the minimum Euclidean distance in a normalized dose‐distance space.^10^ The gamma passing rate (GPR) considers both DD and DTA, providing a measure of the magnitude of the discrepancies between two dose points. This approach is widely used in radiation therapy; however, several research groups have reported limitations of gamma evaluation.^11^, ^12^, ^13^, ^14^, ^15^, ^16^, ^17^, ^18^ Although gamma evaluation can indicate the locations of errors, it does not provide information on the type of error (whether it is a dose or distance error). For example, a gamma value of 2 could indicate a DD of 6.0% in a low‐dose gradient region or a DTA of approximately 4.0 mm in a steep dose gradient region, given a criterion of 3%/2 mm.

In a recent study, Peng et al. applied the structural similarity (SSIM) algorithm to PSQA,^19^ investigated the parameters of the SSIM algorithm and emphasized the importance of the SSIM map. They examined three elements of the SSIM index in relation to variations in the constant values of the SSIM algorithm, providing insights into the correlation between dose errors, dose gradients, distance errors, and the SSIM index. Additionally, the analysis included the picket fence test and clinical cases studies. However, the study did not discuss a meaningful SSIM index for clinical use, nor it provide a consistent criterion for determining pass or fail in clinical cases.

**FIGURE 1:**
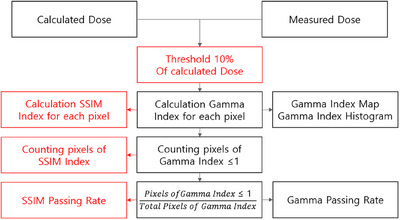
Workflow for quality assessment of radiation therapy plans using gamma Evaluation and SSIM analysis. These red boxes highlight the main tasks of this study.

Figure 1 presents the workflow for assessing the quality of a radiation therapy plan using both gamma evaluation and SSIM analysis. This diagram contrasts the two methods and details the steps involved in each process. The “Calculated Dose” refers to the dose distribution generated by the TPS, while the “Measured Dose” is obtained using devices such as an electronic portal imaging device (EPID). To enhance the accuracy of the analysis, a 10% threshold of the maximum dose is applied to both the calculated and measured dose distributions, excluding regions with doses below this threshold and focusing on the areas with higher doses. For gamma evaluation, the GPR is determined as the percentage of pixels with a gamma index less than or equal to one, where a higher GPR indicates better agreement between the two dose distributions. Similarly, in SSIM analysis, the SSIM index is calculated after excluding regions with doses below the 10% threshold. The SSIM passing rate (SPR) is then defined as the percentage of pixels with SSIM index above the SSIM index threshold, indicating a high level of SSIM between the calculated and measured dose distributions. However, as Wang et al. noted, the SSIM index value required to determine image similarity is not clearly defined.^20^

This study aims to introduce the SSIM algorithm and optimize it as a supplementary metric for clinical cases. We researched the application of the SSIM algorithm in conjunction with commonly used criteria for gamma evaluation, specifically the 3%/2 mm criterion with a 10% threshold, as recommended by the American Association of Physicists in Medicine Task Group (AAPM TG) ‐ 218.^21^ Additionally, we established meaningful criteria for clinical significance to develop innovative supplementary metrics.

## 2 METHODS

Five patients with varying target sizes ranging from 5.0 × 5.0 cm^2^ to 23.0 × 23.0 cm^2^ were selected from cases involving the neck, brain, spine, and breast regions. To ensure the broad applicability of the new metrics, a diverse range of target sizes was deliberately selected. Radiation treatment plans were calculated for these patients’ CT scans. An EPID(a‐Si 1000) mounted on a linear accelerator (LINAC, Clinac iX, Varian Medical System, Palo Alto, CA, USA) was used. The pixel spacing of the dose distribution calculated on the EPID by the TPS (Eclipse, Varian Medical System, Palo Alto, CA, USA) was 0.79 mm, with a pixel count of 384 × 512. All data were analyzed using Python (version 3.11.6) with the Numpy (1.26.1) and TensorFlow (2.14.0) libraries.

### 2.1 DTA algorithm

The SSIM algorithm is a critical tool for image quality assessment that evaluates images based on three components: luminance, contrast, and structure.^21^ The luminance represents the intensity of objects through pixel values in an image, whereas the contrast indicates differences or variations in the luminance. The structure indicates the correlation between the luminance of two images. The comparison function between the images *x* and *y* is defined as follows:

(1) $$\begin{array}{ccc} \mathrm{SSIM}\;\left(x,y\right) & = & l\left(x,y\right)\;\times \;c\left(x,y\right)\;\times \;s\left(x,y\right)\\ & & =\frac{\left(2\mu_{x}\mu_{y}+C_{1}\right)\left(2\sigma_{xy}+C_{2}\right)}{\left({\mu_{x}}^{2}+{\mu_{y}}^{2}+C_{1}\right)\left({\sigma_{x}}^{2}+{\sigma_{y}}^{2}+C_{2}\right)}. \end{array}$$

where *l(x, y)*, *c(x, y)*, and *s(x, y)* represent the luminance, contrast, and structure, respectively, and are calculated as follows

(2) $$l\left(x,y\right)=\frac{2\mu_{x}\mu_{y}+C_{1}}{{\mu_{x}}^{2}+{\mu_{y}}^{2}+C_{1}}.$$

(3) $$c\left(x,y\right)=\frac{2\sigma_{x}\sigma_{y}+C_{2}}{{\sigma_{x}}^{2}+{\sigma_{y}}^{2}+C_{2}}.$$

(4) $$s\left(x,y\right)=\frac{\sigma_{xy}+C_{3}}{\sigma_{x}\sigma_{y}+C_{3}}.$$

Here, $\mu_{x}$ and $\mu_{y}$ represent the mean values of the images *x* and *y*, respectively, while $\sigma_{x}$ and $\sigma_{y}$ denote the standard deviations of images *x* and *y*, respectively. $\sigma_{\mathrm{xy}}$ represents the covariance between the images *x* and *y*. The constants *C_1_*, *C_2_*, and *C_3_* are defined as $C_{1}={\left(K_{1}L\right)}^{2}$, $C_{2}={\left(K_{2}L\right)}^{2}$, with default values $K_{1}=0.01$ and $K_{2}=0.03$, and $C_{3}=C_{2}\;/2$, as specified by Peng et al.^19^ Peng et al. compared these default values with $K_{1}=0.1$ and $K_{2}=0.3$, noting that the response with large *K* and *L* settings is smaller compared to response with smaller *K* and *L* settings.^19^ Thus, we set *K* values to the default as per Wang et al., even though the response of the large *K* and *L* settings might exclude very small dose and gradient disagreements. *L* represents the dynamic range, indicating the range of values in the image. The $\mu_{x}$, $\sigma_{x}$, and $\sigma_{xy}$ are calculated as follows:

(5) $$\mu_{x}=\frac{1}{N}\sum_{i=1}^{N}x_{i}.$$

(6) $$\sigma_{x}={\left(\frac{1}{N-1}\sum_{i=1}^{N}{\left(x_{i}-\;\mu_{x}\right)}^{2}\right)}^{1/2}.$$

(7) $$\sigma_{xy}=\frac{1}{N-1}\sum_{i=1}^{N}\left(x_{i}\;-\;\mu_{x}\right)\left(y_{i}\;-\;\mu_{y}\right).$$

Here, *i* represents the index of all points within the region, and *N* is the total number of points within the same region. The SSIM index was calculated by combining the three components: luminance, contrast, and structure. The SSIM index ranges between 0 and 1, with values closer to 1 indicating greater similarity between the two images. A localized comparison using a window of size N×N is more effective than a comparison of the entire image at once. This is because distortions and statistical characteristics of images often manifest locally rather than uniformly across the entire image, facilitating a more detailed analysis of local characteristics. For the SSIM analysis, the dose distributions of the reference field and errors were designated as images *x* and *y*, respectively, according to Equation (1). The default values of the constants *K_1_* and *K_2_* were used, with L set as the maximum value a pixel can have. For 8‐bit grayscale images, Wang et al. used *L* = 255, while Peng et al. used *L* = 200 for a conventional fraction dose.^19^, ^20^ In our study, we set *L* value as the maximum values of each dose distribution. Since each dose distribution has a different maximum value, we did not set the *L* value as a constant. Additionally, to account for local characteristics, the window size was set to 4×4 pixels with a pixel spacing of 0.79 mm. The filter used in SSIM is a gaussian filler with a width of 1.5. This size corresponds to the pixel spacing of the fluence map, which is 2.37 mm, for subsequent comparison with the gamma evaluation.

### 2.2 Influence of the distance and dose errors on the GPR and SSIM index

We investigated the effect of variations in the distance and dose errors on the GPR and SSIM index to assess the performance of the PSQA for intensity modulated radiation therapy (IMRT) fields and volumetric‐modulated arc therapy (VMAT), as calculated using the TPS. We generated dose distributions for 40 fields that would be measured using the EPID. The dose distributions ranged approximately from $6.0\times 6.0\;cm^{2}$ to $24.0\times 24.0\;cm^{2}$ and distance and dose errors were introduced. The distance errors were adjusted within the range of −5 mm to +5 mm, while the dose errors varied from 0.95 to 1.05. Distance errors were adjusted only along the *x*‐axis, as they affect gamma evaluation irrespective of direction. The dose distributions were shifted by −5 mm to +5 mm in increments of 0.01 mm using Python. Dose errors, considered anticipated errors due to factors such as output variations or EPID calibration, were adjusted as scale factors. The dose distributions were multiplied by scale factors ranging from 0.95 to 1.05 in increased of 0.01 using Python. The dose distributions were calculated for various dose and distance errors across all 40 fields. To analyze the relationship between the GPR and SSIM index for each error condition, we computed the Pearson correlation coefficient (PCC). The PCC measures the linear relationship between two datasets, standardized by the product of their covariance and standard deviations.

(8) $$r_{XY}=\frac{\sum_{i}^{n}\left(X_{i}-\bar{X}\right)\;\left(Y_{i}-\bar{Y}\right)}{\sqrt{\sum_{i}^{n}{\left(X_{i}-\bar{X}\right)}^{2}}\sqrt{\sum_{i}^{n}{\left(Y_{i}-\bar{Y}\right)}^{2}}}\;$$

Therefore, the PCC ranges from −1 to 1. A value close to 1 indicates a strong positive linear correlation, meaning that the metric increases with the errors. Conversely, a value close to −1 indicates a strong negative linear correlation, suggesting that the metric decreases with increasing errors. Additionally, a value close to 0 indicates no linear correlation between the errors and the metric. Thus, this analysis allowed us to quantitatively examine the linear correlation between the errors and the two evaluation metrics.

### 2.3 SPR

Gamma evaluation is typically performed using a 10% threshold while the SSIM algorithm calculates values across all regions. In areas with no dose, the SSIM index tends to approach 1. To refine the SSIM index results, we defined a region of interest (ROI) by excluding areas in the reference dose distribution where the dose was less than 10% of the maximum dose. A GPR represents the percentage of pixels with a gamma index less than or equal to 1 in regions exceeding the 10% threshold. A gamma index closes to 0 indicates similarity between the two dose distributions. Similarly, we introduced the SPR as the percentage of pixels with an SSIM index close to 1 in regions above the 10% threshold. A higher SSIM index indicated greater similarity between the two dose distributions.

However, as Wang et al. noted, the SSIM index value required to determine images similarity is not clearly defined.^20^ To address this, we identified an appropriate SSIM index valid value for this study. When the distance error is within 2 mm, the GPR with a 2 mm criterion is 100%, but it drops below 100% when the error exceeds 3 mm. Following this logic, we tested SSIM index thresholds from 0.00 to 1.00 in increments of 0.01 to determine the SSIM index valid value at which the SPR is 100% at 2 mm and decreases below 100% at 3 mm.

### 2.4 Application of the SPR to clinical cases

In this study, we aimed to use SPR as a supplementary metric for Gamma Evaluation. We calculated both the GPR(3%/2 mm) and the SPR for 40 fields. These fields, with target sizing from $5.0\times 5.0\;cm^{2}$ to $23.0\times 23.0\;cm^{2}$, were measured using an EPID. These fields were from neck, brain, spine, and breast. To ensure broad applicability of the new metric, we selected a diverse range of target sizes and used the 3%/2 mm criterion for GPR, which is commonly used for IMRT or VMAT. Our goal is for SPR which represents the percentage of pixels with an SSIM index above a valid value to be applicable to all PSQA like GPR as recommended by AAPM TG‐218. Therefore, we applied SPR to targets of various sizes rather than limiting it to specific sites. We particularly focused on fields where the GPR with the 3%/2 mm criterion was less than 95%. This allowed us to distinguish whether failure in these cases were due to distance or dose errors.

## 3 RESULTS

### 3.1 Correlation analysis of GPR and SSIM index according to errors

**FIGURE 2:**
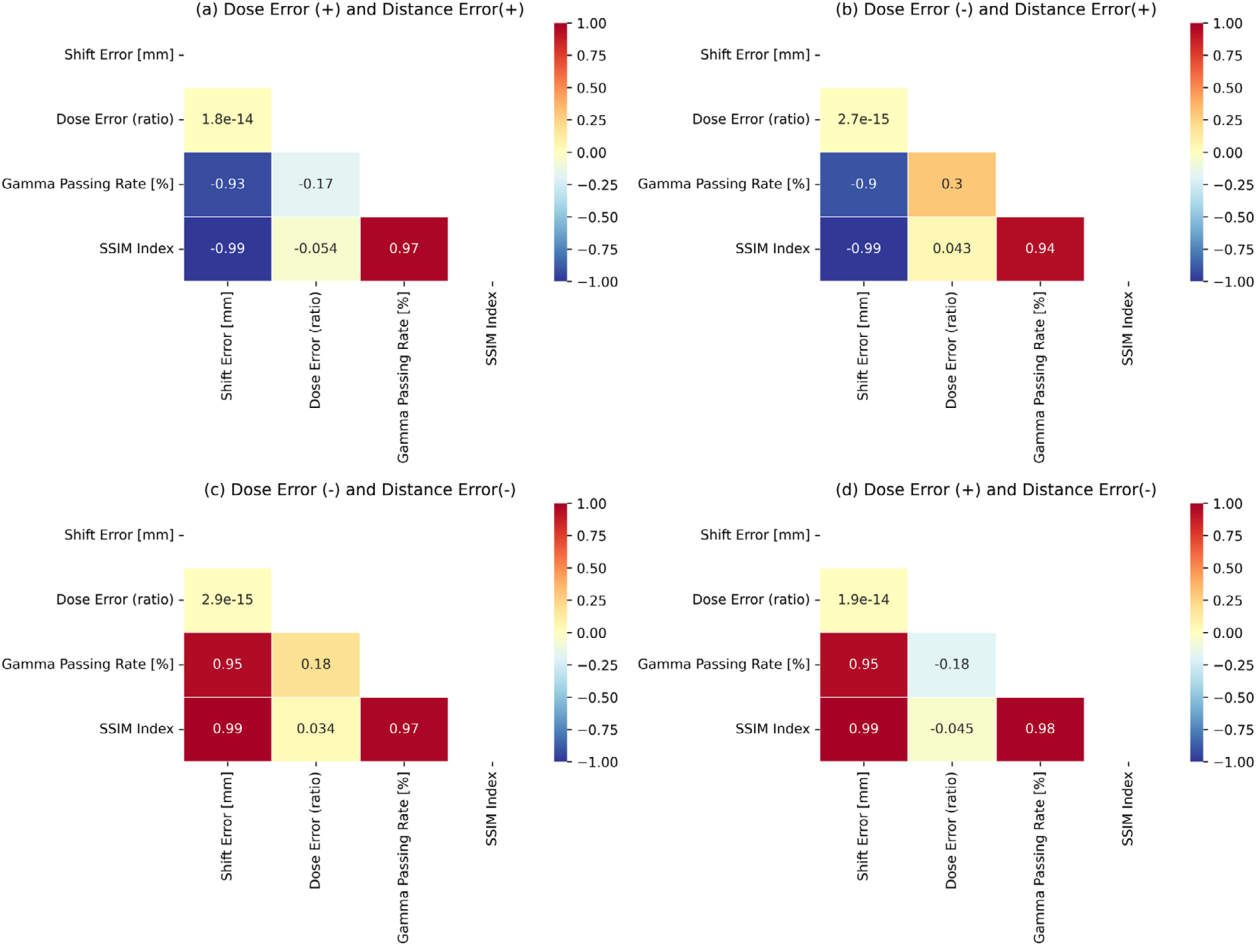
PCCs of the GPR and SSIM index according to the distance and dose errors. (a) Dose error positive and distance error positive. (b) Dose error negative and distance error positive. (c) Dose error negative and distance error negative. (d) Dose error positive and distance error negative. GPR, gamma passing rate; PCC, Pearson Correlation Coefficient; SSIM, structural similarity.

We investigated the impact of variations in the distance error and dose error on the GPR and SSIM index by varying the distance error from −5 mm to +5 mm and the dose error from 0.95 to 1.05 for all 40 fields using Python. The dose distributions were shifted by −5 mm to +5 mm in increments of 0.01 mm and multiplied by scale factors ranging from 0.95 to 1.05 in increments of 0.01 using Python. We calculated the dose distributions for the dose errors and distance errors for each of 40 fields. Figure 2 shows the PCC distribution of GPR and SSIM index values for each error condition. The GPR decreases as both distance and dose errors increase. In contrast, the SSIM index decreases with increasing distance error, while the effect of the dose error is relatively lower. A detailed analysis examined correlations based on the direction of the distance and dose errors. Distance error has a relatively greater impact on both GPR and SSIM index, whereas dose error has minimal effect on GPR and almost no effect on the SSIM index.

#### 3.1.1 Positive distance error and positive dose error

When both distance and dose errors were positive, the results quantitatively illustrated the linear relationships between these errors, the GPR, and the SSIM index through the PCC. As the distance error increased positively, the PCC value with GPR was −0.93. As the dose error increased positively, the PCC value with GPR was −0.17. In contrast, as the distance error increased positively, the PCC value with the SSIM index was −0.99. When the dose error increased positively, the PCC value with the SSIM index was −0.054. Furthermore, when both distance and dose errors increased positively, the PCC between GPR and SSIM index was 0.97.

#### 3.1.2 Positive distance error and negative dose error

When the distance error was positive and dose error was negative, the results quantitatively illustrated the linear relationships between these errors, the GPR, and the SSIM index through the PCC. As the distance error increased positively, the PCC value with GPR was −0.9. As the dose error increased negatively, the PCC value with GPR was 0.3. In contrast, As the distance error increased positively, the PCC value with SSIM index was −0.99. When the dose error increased negatively, the PCC with SSIM index was 0.043. Furthermore, when the distance error was positive and the dose error was negative, the PCC between the GPR and SSIM index was 0.94.

#### 3.1.3 Negative distance error and negative dose error

When both the distance and dose errors were negative, the results quantitatively illustrated the linear relationships between these errors, GPR, and SSIM index through the PCC. As the distance error decreased negatively, the PCC value with GPR was 0.95. As the dose error decreased negatively, the PCC value with GPR was 0.18. In contrast, as the distance error decreased negatively, the PCC value with the SSIM index was 0.99. When the dose error decreased negatively, the PCC for the SSIM index was 0.034. Furthermore, when both the distance and dose errors decreased negatively, the PCC between the GPR and SSIM index was 0.97.

#### 3.1.4 Negative distance error and positive dose error

When the distance error was negative and dose error was positive, the results quantitatively illustrated the linear relationships between these errors, the GPR, and the SSIM index through the PCC. As the distance error decreased negatively, the PCC value with GPR was 0.95. As the dose error increased positively, the PCC value with GPR was −0.18. In contrast, as the distance error decreased negatively, the PCC value with SSIM index was 0.99. When the dose error increased positively, the PCC value with SSIM index was −0.045. Furthermore, when the distance error was negative and dose error was positive, the PCC between the GPR and SSIM index was 0.98.

### 3.2 SPR

#### 3.2.1 Comparative analysis of the SSIM index before and after thresholding

**FIGURE 3:**
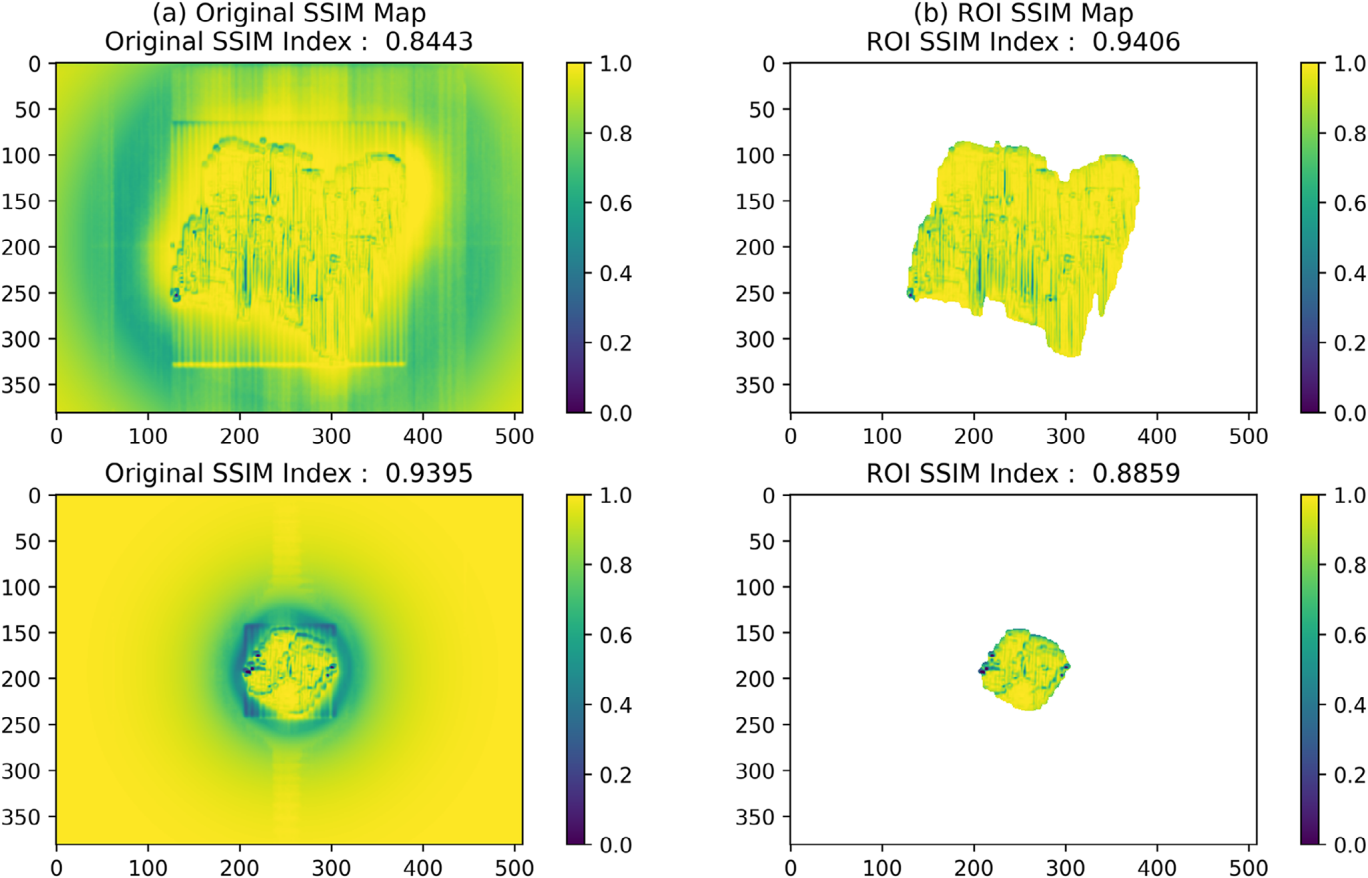
The example comparison of the SSIM maps before and after setting the threshold. (a) The original SSIM maps without ROI. (b) The calculated SSIM maps with ROI. The above was increased SSIM index but the below was decreased or increased SSIM index after thresholding by scatter. ROI, region of interest; SSIM, structural similarity.

We quantitatively investigated the relationships between the SSIM index and the errors. We confirmed that the SSIM index was not significantly affected by dose errors but was primarily influenced by distance errors. Theoretically, the SSIM algorithm computes values for all regions, and areas with no dose should have an SSIM index close to 1. However, due to the scattered radiation outside the ROI in measured dose distributions, the SSIM index is not always 1. For instance, as shown in upper image of Figure 3a, a point dose outside of the ROI in the TPS is 0, whereas the same point in the measurement is not 0, resulting in an SSIM index below 1. Conversely, in lower image of Figure 3a, when the point dose outside the ROI in both the TPS and the measurement is close to 0, the SSIM index is nearly 1. These examples illustrate that the SSIM index cannot be reliably predicted outside the ROI. To address this issue, we defined the ROI by applying a threshold of 10%, consistent with the GPR criteria. This excluding areas with doses less than 10% of the maximum dose in the reference dose distribution. Before applying the threshold, the SSIM index ranged from 0.7772 to 0.9842 across the 40 fields. After thresholding, this range shifted to 0.8859 to 0.9588. These changes are primarily due to SSIM indices outside the ROI approaching 1 or other values because of the background scatter. Setting the threshold allowed us to quantitatively assess the distance errors at the same locations used in the gamma evaluation.

#### 3.2.2 Exploring the valid criterion for the SSIM index

**FIGURE 4:**
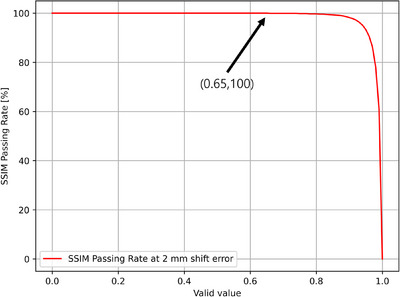
SSIM index valid values. Graph illustrating the valid values for a distance error of 2 mm. The SPR remains at 100% for SSIM index values up to 0.65 but begins to decrease for values above 0.65. This graph helps determine the appropriate SSIM index criterion for assessing the validity of the SPR. SPR, structural similarity passing rate; SSIM, structural similarity.

As observed in Figure 3, while assessing the SSIM index relation to distance errors, the criteria for determining acceptable levels were not well‐defined. To address this, we introduced a concept similar to the GPR. We analyzed the SSIM index by calculating the percentage of pixels with an SSIM index close to 1 within the ROI defined by a threshold of 10% or more of the maximum dose. However, as previously noted, the specific valid SSIM index values for assessing image quality were not clear. To establish an appropriate criterion, we evaluated the percentage of pixels with an SSIM index above various value from 0.00 to 1.00 in increments of 0.01, calculating the SPR for each value. For comparison, the GPR remains at 100% for a distance error of 2 mm but drops below 100% for a distance error of 3 mm. Given that the SPR is primarily influenced by distance errors, we assessed its behavior at distance error of 2 and 3 mm. The SPR should ideally be 100% for a distance error of 2 mm and decreased below 100% for a distance error of 3 mm. As shown in Figure 4, an SSIM index criterion of 0.65 exhibited the desired behavior: It resulted in an SPR of 100% but decrease to 99.9% for values above this criterion. Thus, we propose setting the valid SSIM index criterion at 0.65. This criterion ensures that if the SPR value is less than 100%, indicates the presence of pixels with distance errors greater than 2 mm within the ROI.

### 3.3 Application of the SPR to clinical cases

**FIGURE 5:**
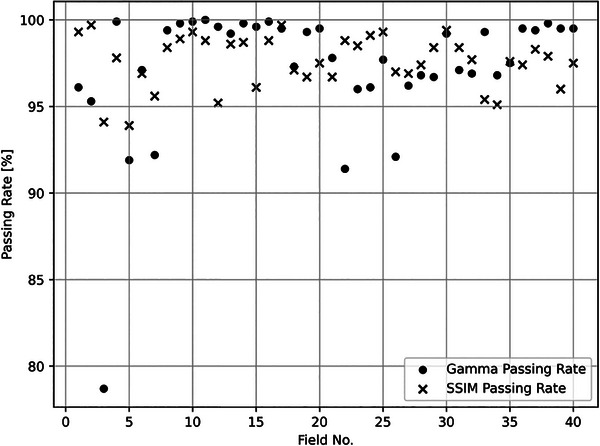
Displays the comparison between GPR and SPR values across the 40 fields. The GPR values ranged from 78.7% to 100%, while the SPR values varied from 93.9% to 99.7%, Notably, there is a discrepancy between the SPR and GPR values for different fields. GPR, gamma passing rate; SPR, structural similarity passing rate.

We calculated both the GPR with a 3%/2 mm criterion and SPR with a threshold of ≥ 0.65 for 40 clinical fields. These fields, measured by EPID, included cases from the neck, brain, spine, and breast regions. Our aim was to evaluate the global applicability of the new metrics, and the results are depicted in Figure 5.

As discussed earlier, the SPR represents the percentage of pixels with distance error within 2 mm relative to the ROI. Thus, a lower SPR value indicates a higher frequency of distance errors within the ROI. Specifically, among the five fields with GPR values below 95%, the GPR values were 78.7%, 91.9%, 92.2%, 91.4%, and 92.1%, respectively. The corresponding SPR values for these fields were 94.1%, 93.9%, 95.6%, 98.8% and 97.0%. This comparison illustrates that while SPR and GPR values share similar trends, they do not always align. Both metrics tend to decrease in the presence of distance errors, but they can yield different results. This suggests that while the SPR and GPR metrics reflect related aspects of dose distribution accuracy, they provide complementary information for may highlight different issues in quality assurance.

## 4 DISCUSSION

In this study, we utilized the SSIM algorithm to detect discrepancies between two dose distributions and investigate the impact of variations in distance and dose errors on both the GPR and SSIM index. Our findings revealed distinct relationships between the types of errors on the response of both metrics, providing insights into their utility for PSQA.

Our analysis indicated that both GPR and SSIM index were negatively correlated with an increase in positive distance errors. Conversely, with negative distance errors, both GPR and SSIM index showed a positive correlation. This consistent correlation, regardless of the direction of the distance error, suggests that both metrics are similarly affected by changes in spatial distribution. In contrast, the effect of dose errors on these metrics was different. The GPR showed a negative correlation with positive dose errors and a positive correlation with negative dose error, implying that GPR is sensitive to both the magnitude and direction of dose discrepancies. However, the SSIM index remained largely unaffected by dose errors in either direction, highlighting that it primarily responds to spatial discrepancies rather than dose magnitude differences.

To address the lack of established criteria for SSIM index values in assessing dose distribution equivalency, we introduced the SPR, akin to GPR. By defining the ROI with a threshold of 10% of the maximum dose, we aligned the SSIM evaluation with gamma evaluation locations. The SPR was then defined as the percentage of pixels with an SSIM index above the SSIM index threshold (≥0.65). This threshold was selected to ensure SPR was 100% at a 2 mm distance error and decreased below 100% at a 3 mm error, thus mirroring the behavior of GPR under similar conditions. Applying SPR to 40 clinical fields, we observed that the range of SPR values (93.9%–99.7%) was narrower than that of GPR (78.7% to 100%). This narrower range does not imply less sensitivity; instead, it reflects that SPR captures structural similarities, focusing on the spatial distribution quality rather than solely on dose agreement. Therefore, SPR might offer a more robust evaluation in clinical scenarios where structural alignment is more critical than dose accuracy alone.

We specifically analyzed cases with GPR values below 95% to differentiate between the types of errors contributing to the failure. When the SPR value was low (e.g., the first and second fields with SPR values of 94.1% and 93.9, respectively), it indicated predominant distance errors, likely due to mechanical issues such as multileaf collimator (MLC) misalignment or treatment machine mechanical problems. Conversely, when SPR values were close to 100% (e.g., third, fourth, and fifth fields with SPR values of 95.6%, 98.8%, and 97.0%), the discrepancies were mainly due to dose errors, potentially related to issues like low monitor unit segments or output inconsistencies. In clinical practice, if multiple PSQA measurements indicate GPR values below 95% on a single day, analyzing SPR can help distinguish between dose‐related and distance‐related errors. High SPR values suggest the need for dose‐related QA, such as recalibrating measurement tools or verifying the linear accelerator output. Low SPR values, on the other hand, point toward mechanical QA checks, including assessments of the collimator and MLC.

**FIGURE 6:**
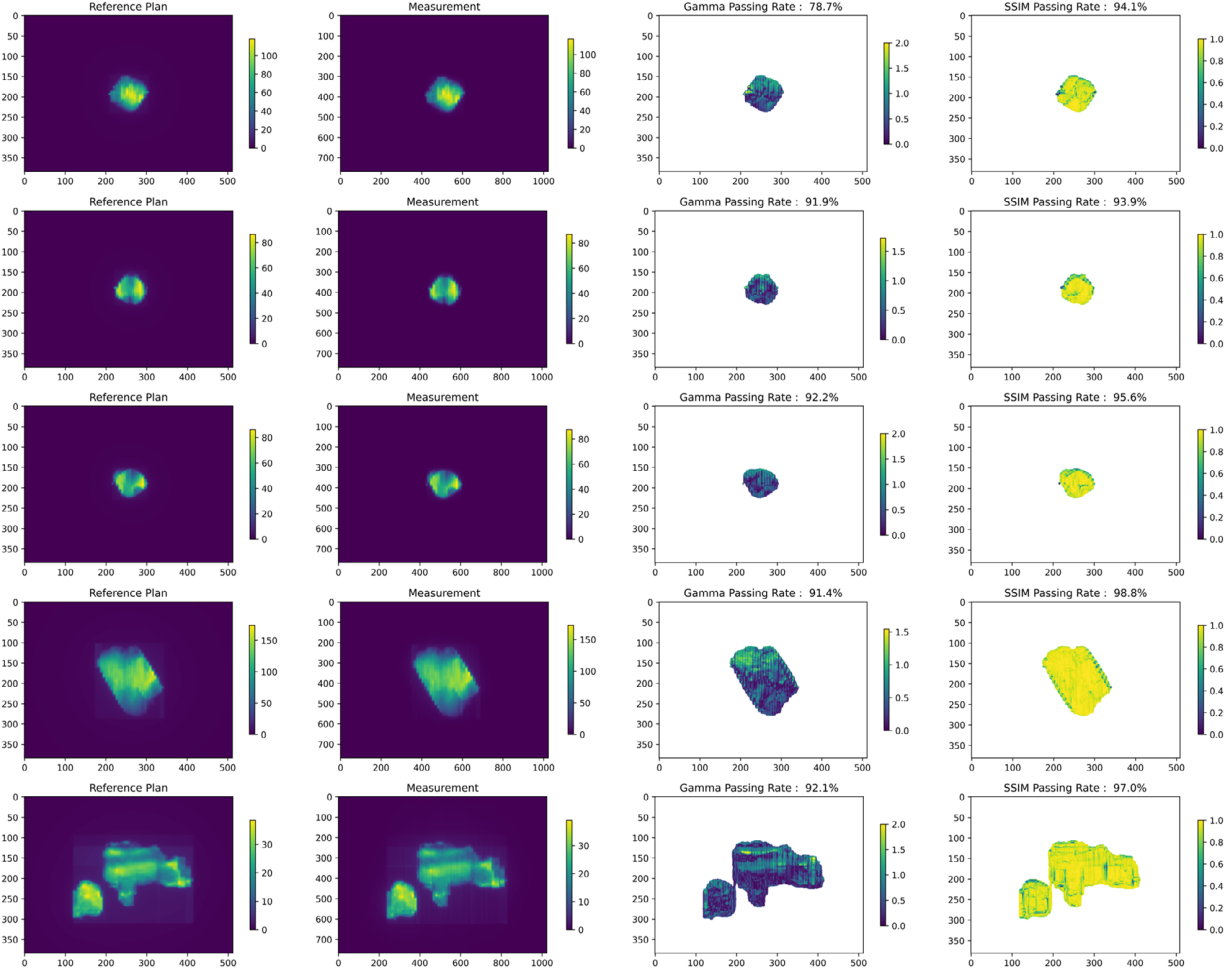
Analysis of dose distribution maps for five fields with GPR values below 95%. The figure includes four maps for each field, presented from left to right: Reference (Calculated) dose, Measurement Dose, Gamma map (3%/2 mm), SSIM map (≥0.65). The SSIM map showing regions where the SSIM index meets or exceeds the threshold of 0.65, indicating structural similarity. GPR, gamma passing rate; SSIM, structural similarity.

Unlike the DD and DTA methods, which focus solely on dose magnitude and spatial agreement, respectively, the combination of GPR and SPR provides a more comprehensive error analysis. DTA, while effective in regions with steep dose gradients, may not perform well in shallow gradient areas and is challenging to interpret visually. SPR, calculated using a 2 mm criterion, applies effectively across both steep and shallow gradient regions, providing consistent insight. The SSIM maps, as show in Figure 6, clearly highlighted area with significant distance error, offering a visual tool for error localization.

This study demonstrated that SPR offers valuable supplementary information to GPR, enhancing the evaluation of PSQA, However, it is not intended to replace established metrics like gamma index, DD, or DTA. Each metric has its limitations, but when used in conjunction with SPR, a more detailed understanding of PSQA failures is achievable. Future research will explore the effects of varying constant values ($K_{1}$, $K_{2}$, L) on SPR to refine its sensitivity and applicability. Moreover, while SPR provides insights into the types of errors (distance vs. dose), it does not pinpoint their specific sources. With recent developments in log‐based PSQA methods, which address uncertainties in spatial delivery and MLC performance, future studies will investigate the correlation between these methods and SPR for a more holistic approach to PSQA.

In conclusion, the incorporation of SPR alongside traditional GPR metrics enhances the ability to identify and characterize discrepancies in dose distributions, ultimately improving the accuracy and safety of radiation therapy treatments.

## 5 CONCLUSIONS

In this study, we introduced the SPR to establish criteria for the SSIM index, identifying scenarios where the SPR falls below 100% at a distance error of 3 mm. This led to a crucial finding: SPR can be effectively used to quantitatively analyze the impact of distance errors. By applying SPR to clinical cases, we examined failed instances and compared the GPR with SPR. Our findings indicate that while the GPR is unable to differentiate between distance and dose errors, SPR provides valuable insights by identifying causes related to distance errors when used as a supplementary metric. This makes SPR a valuable tool for interpreting PSQA results.

While we proposed SPR as an enhanced evaluation metric for PSQA using the SSIM algorithm, various avenues for future research remain. Further studies are required to assess the performance of the developed SPR metrics across different dose distributions and criteria verifying their generalizability and stability. Additionally, exploring the effectiveness of these methods in diverse clinical environments using different radiation treatment machines and measurement tools will provide insights into the broader applicability of SPR.

This study demonstrated the combined utility of GPR and SPR in identifying and resolving the causes of dose and distance errors, indicating potential applications in clinical settings. The methods proposed and results obtained are expected to improve quality and patient safety in the field of radiation therapy, providing a solid foundation for future PSQA evaluations and casual analyses. As such, the integration of SPR into routing PSQA practices could significantly enhance the precision and reliability of radiation therapy, ultimately contributing to better patient outcomes.

## AUTHOR CONTRIBUTIONS

Jae Choon Lee conceived the concept, analyzed the data, wrote the draft, and edited the final manuscript. Hyeong Wook Park acquired and analyzed the data and edited the manuscript. Young Nam Kang conceived the concept, supervised the study, and edited the final manuscript. All authors have read and agreed to the published version of the manuscript.

## CONFLICT OF INTEREST STATEMENT

The authors declare no conflicts of interest.
